# Federated learning for privacy-preserving ophthalmic artificial intelligence: clinical applications and translational challenges

**DOI:** 10.3389/fmed.2026.1904004

**Published:** 2026-07-29

**Authors:** Yuan Wei, Kaikai Zhao, Andrzej Grzybowski, Kai Jin

**Affiliations:** 1 Eye Center of Second Affiliated Hospital, School of Medicine, Zhejiang University, Hangzhou, Zhejiang, China; 2 Zhejiang Provincial Key Laboratory of Ophthalmology, Zhejiang Provincial Clinical Research Center for Eye Diseases, Zhejiang Provincial Engineering Institute on Eye Diseases, Hangzhou, Zhejiang, China; 3 School of Computer Science and Technology/School of Artificial Intelligence, China University of Mining and Technology, Xuzhou, China; 4 Department of Ophthalmology, University of Warmia and Mazury, Olsztyn, Poland; 5 Foundation for Ophthalmology Development, Institute for Research in Ophthalmology, Poznań, Poland

**Keywords:** data security, federated learning, ophthalmology, optical coherence tomography, retinal imaging

## Abstract

Federated learning (FL) is increasingly relevant to ophthalmology because retinal photographs, optical coherence tomography (OCT), OCT angiography, visual fields, and linked clinical records are clinically valuable but difficult to pool across institutions. In this narrative review, we synthesize ophthalmology-focused FL literature across diabetic retinopathy (DR), glaucoma, age-related macular degeneration (AMD), pediatric retinal disease, multi-disease retinal diagnostics, and emerging ophthalmic platforms. Current evidence suggests that FL can support collaborative AI development without centralizing raw patient data, and selected studies show performance close to centralized training under controlled retrospective or multicenter experimental conditions. For example, multicenter glaucoma detection from volumetric OCT achieved an AUC of 0.92 with FL compared with 0.94 for centralized training. However, FL is privacy-enhancing rather than privacy-complete, and most ophthalmic FL systems have not yet undergone prospective clinical validation. Model updates may remain vulnerable to gradient inversion, membership inference, poisoning, site-level bias, and latent identity or attribute leakage. For eye-care networks, the main value of FL is therefore not simply algorithmic performance but a governance model for privacy-conscious collaboration. Prospective validation, interoperability, explainability, workflow integration, privacy auditing, and clear responsibility for monitoring are needed before FL-enabled ophthalmic AI can be deployed routinely.

## Introduction

Artificial intelligence (AI), particularly deep learning, has reshaped ophthalmic image analysis in diabetic retinopathy, glaucoma, age-related macular degeneration, and other sight-threatening conditions (1, 2). However, clinically reliable AI still depends on access to diverse, well-annotated, and geographically representative datasets (3). In routine practice, that requirement conflicts with the way ophthalmic data are actually held: images and clinical records remain fragmented across institutions and are constrained by privacy legislation, local governance, contractual obligations, and commercial concerns (4).

Centralized data pooling is therefore often impractical in ophthalmology. Retinal images, OCT volumes, and linked clinical metadata may contain both direct identifiers and latent re-identification risk. Cross-border data transfer is further limited by legal, ethical, and institutional requirements, and poor harmonization between datasets may weaken both generalizability and fairness (5, 6).

FL provides a principled alternative. Rather than moving data to a central repository, it sends a model to each participating site for local training and aggregates only model updates or intermediate representations (7). This “data stay, model move” paradigm reduces the need for raw data sharing while enabling collaboration across hospitals, regions, and countries. FL alone does not guarantee privacy, but it creates the architectural basis on which secure aggregation, differential privacy, and homomorphic encryption can be layered (8).

The relevance of FL to ophthalmology has become increasingly apparent since approximately 2021, when the literature began to move from broad conceptual discussion toward application-specific studies in DR, glaucoma, AMD, OCT/OCTA analytics, platform development, and security-oriented investigation (9, 10). At the same time, recent work has shown that model updates may still leak sensitive image information, underscoring the need for rigorous privacy engineering beyond simple federated averaging (11).

In this narrative review, we synthesize the current landscape of FL in ophthalmic AI with an emphasis on clinical relevance. The review was informed by targeted literature screening of ophthalmology, medical imaging, and digital health publications addressing federated learning, privacy-preserving computation, and collaborative ophthalmic AI. Because this was a narrative review rather than a systematic review, the available evidence was synthesized qualitatively and no pooled estimates were calculated. We first summarize the FL paradigms most relevant to eye care, then review disease-specific applications, and finally discuss the privacy, governance, and implementation issues that will determine whether these systems can move from proof-of-concept studies into clinical use. Compared with prior broad reviews of privacy-preserving ophthalmic AI, this review emphasizes disease-specific translational barriers, ophthalmology-specific privacy risks, quantitative context where available, and the distinction between benchmark performance, clinical utility, and real-world deployment readiness.

### Literature search approach

To inform this narrative review, we performed targeted literature screening in PubMed, Web of Science, and Google Scholar for publications addressing federated learning, privacy-preserving computation, and collaborative ophthalmic artificial intelligence. Search terms included combinations of “federated learning,” “ophthalmology,” “retina,” “diabetic retinopathy,” “glaucoma,” “age-related macular degeneration,” “retinopathy of prematurity,” “optical coherence tomography,” “OCT angiography,” “privacy,” “secure aggregation,” “homomorphic encryption,” and “differential privacy.” The search was conducted initially in March 2026 and included publications available online from January 2014 to March 2026.

Eligible publications included peer-reviewed original studies, platform papers, methodological papers, systematic or narrative reviews, and clinically relevant privacy or governance articles that directly informed ophthalmic FL. We prioritized studies involving ophthalmic imaging, multicenter or cross-silo learning, external validation, privacy-preserving computation, domain shift, fairness, implementation, or clinical translation. We excluded papers that addressed general FL without medical or ophthalmic relevance, articles without sufficient methodological detail, preclinical examples that did not inform ophthalmic deployment, and duplicate reports of the same system unless they contributed distinct technical or clinical information.

Study selection was therefore purposive rather than exhaustive. Titles, abstracts, and full texts were screened for relevance by the authors, and reference lists of key articles were reviewed to identify additional studies. Because no formal systematic review protocol was registered, no PRISMA flow diagram or pooled effect estimate was produced. This design supports clinically oriented synthesis but introduces possible selection bias, which is addressed explicitly in the Limitations section. Additional details are provided in Supplementary Tables 1, 2.

## Clinical rationale and federated learning paradigms in ophthalmology

### Core FL workflow

**FIGURE 1:**
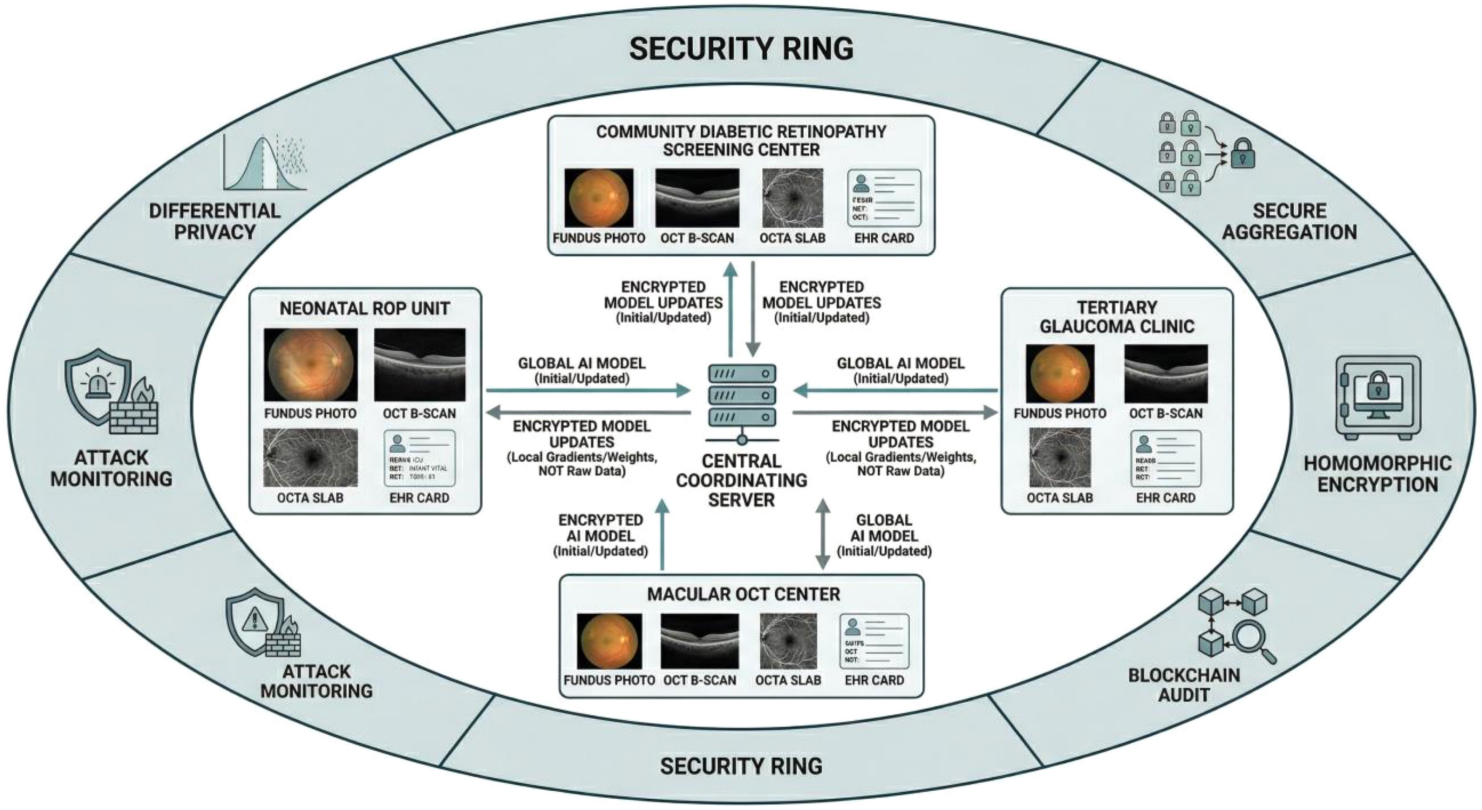
Conceptual architecture of privacy-preserving federated learning in ophthalmology. This conceptual, illustrative schematic shows multiple ophthalmic institutions (screening center, tertiary eye hospital, academic center, neonatal unit) each retaining local retinal photographs, OCT/OCTA scans, and structured clinical data. It is not derived from a single real-world deployment. A global model is distributed from a coordinating server to each site, trained locally, and only encrypted or masked model updates are returned for aggregation. An outer security layer illustrates secure aggregation, differential privacy, homomorphic encryption, and audit logging.

A typical cross-silo FL system includes a coordinating server and several participating institutions. The server initializes a model, distributes it to local sites, and aggregates site-level updates after each training round (12). The most widely used baseline strategy is Federated Averaging (FedAvg), which combines local model parameters according to sample size. In ophthalmology, this arrangement is well suited to hospital-based collaboration, where institutions often collect similar imaging modalities but serve different patient populations. The conceptual architecture of privacy-preserving ophthalmic FL is illustrated in Figure 1.

### Horizontal, vertical, and transfer federated learning

Horizontal FL represents one of the most commonly adopted models in ophthalmic applications. Here, collaborating centers usually work with the same modality, such as fundus photography or OCT, but draw from different patient populations. Vertical FL may become relevant when complementary data are held by different institutions, for example when imaging data are stored in eye hospitals but structured systemic or laboratory variables are held elsewhere (13). However, concrete ophthalmic vertical-FL demonstrations remain sparse. Federated transfer learning may also be useful when overlap in both samples and features is limited, such as in cross-modal collaboration or in networks spanning pediatric and adult eye care. Thus, horizontal federated learning has accumulated the most substantial empirical evidence in ophthalmology to date. In contrast, vertical and transfer federated learning represent promising but still under-explored avenues that require additional validation prior to broader clinical adoption.

### Beyond FedAvg: heterogeneity-aware and personalized FL

Ophthalmic datasets are rarely independent and identically distributed. Variation in disease prevalence, acquisition devices, image quality, ethnicity, referral thresholds, and annotation practice creates marked non-IID conditions (14). This has encouraged the use of approaches such as personalization, self-supervised FL, asynchronous FL, generative replay, and federated adapter tuning (15, 16). These strategies aim to preserve the value of collaboration while improving stability and local clinical relevance (17) but they introduce trade-offs in communication cost, privacy accounting, implementation complexity, and comparability across sites.

### Why federated learning is clinically attractive in ophthalmology

Ophthalmology is particularly well suited to FL because the specialty is image-rich, disease burden is unevenly distributed, and many workflows are screening-based. Retinal photographs, OCT, OCTA, slit-lamp images, ultrasound, and visual fields can all be digitized and analyzed at scale. Yet single centers often lack the diversity needed to build robust models on their own, especially for rare diseases or under-represented populations. FL may therefore help bridge the gap between local data stewardship and broader collaborative model development (4).

## Current clinical applications of federated learning in ophthalmology

### Diabetic retinopathy

DR remains the most extensively studied ophthalmic use case for FL. Early studies showed that OCT-based microvasculature segmentation and DR classification could be trained collaboratively without centralizing data (18). Later work extended FL to fundus-based DR grading and other privacy-conscious classification approaches, including transformer-based models and personalization strategies (19, 20). Multicenter screening-network studies have further increased the translational relevance of DR-focused FL research (21).

The appeal of FL in DR is clinically plausible: screening networks are geographically distributed, disease prevalence and referral thresholds vary across institutions, grading standards differ between programs, fundus cameras are heterogeneous, and the consequences of data leakage are substantial (1). More recent work has also explored encrypted FL pipelines for DR classification, illustrating how privacy-enhancing techniques can be operationalized in ophthalmic practice (22).

### Glaucoma

Glaucoma is another major application area, especially for OCT-based detection and structural assessment. A landmark multicenter study showed that privacy-preserving FL could support glaucoma detection from volumetric OCT scans across international sites while maintaining performance close to centralized approaches (23). In that study, the reported AUC was 0.92 for the federated model compared with 0.94 for centralized training, providing a compelling quantitative example in ophthalmology demonstrating that cross-silo FL can approach centralized performance under controlled multicenter conditions.

Recent literature has also expanded the scope of glaucoma FL beyond simple cross-silo image classification, although differences in OCT vendors, scan protocols, segmentation algorithms, visual-field strategies, and disease-severity distributions remain major barriers to generalizable deployment. Review work has highlighted the relevance of personalized learning, device heterogeneity, domain shift, and intellectual-property considerations in collaborative glaucoma AI (24). Emerging multimodal and vertical FL frameworks suggest that future glaucoma screening may benefit from decentralized fusion of imaging, visual fields, and structured clinical metadata (13).

### Age-related macular degeneration and OCT/OCTA analytics

AMD and related macular diseases are natural targets for FL because OCT and OCTA datasets show substantial site-specific variation. Published work suggests that FL can support AMD diagnosis from OCT images, particularly when combined with domain adaptation to address inter-institutional differences (25). More recently, OCTA-based studies have expanded the field from binary disease detection toward broader retinal classification in federated settings (26).

These studies are important because they move ophthalmic FL beyond fundus photography and into higher-dimensional imaging, where both privacy risk and communication cost may be greater. They also highlight that performance depends not only on the FL algorithm itself, but on alignment of preprocessing, feature representation, signal-strength thresholds, OCT/OCTA artifact handling, segmentation pipelines, vendor-specific metrics, and site-specific disease spectrum (15).

### Retinopathy of prematurity and pediatric ophthalmology

Published FL work in retinopathy of prematurity (ROP) and other pediatric ophthalmic applications remains limited, so this area should be regarded as an emerging and under-explored use case rather than an established application domain. Its inclusion is justified by the concentration of pediatric retinal datasets in specialized centers, uneven case volumes, heightened privacy scrutiny, and the ethical difficulty of broad raw-data pooling. Existing ocular-imaging reviews have repeatedly identified ROP as a clinically relevant setting for privacy-preserving multicenter collaboration, but prospective or task-specific FL evidence remains an important gap (6).

### Multi-disease recognition, ophthalmic platforms, and emerging large-model adaptation

Recent work has moved beyond single-disease models toward broader ophthalmic platforms. FedEYE is an important example, offering a scalable end-to-end FL platform designed specifically for ophthalmology (27). This matters clinically because translation depends not only on benchmark performance, but also on site onboarding, workflow integration, update traceability, quality control, local validation, and the operational burden placed on participating centers.

The field is also beginning to intersect with large-scale pretrained and foundation-model development. It is important, however, to distinguish distributed data-parallel training from cross-institutional FL. Distributed training can coordinate computation across machines or sites but does not necessarily include the governance, client autonomy, privacy accounting, secure aggregation, and site-level control that define clinically realistic FL. Recent ophthalmic foundation-model work is therefore best interpreted as adjacent evidence that large-scale ophthalmic representation learning may benefit from decentralization, not as proof that federated foundation models are deployment-ready (28). Related work in broader diabetes-care multimodal federated AI and in federated adapter tuning suggests a possible path toward parameter-efficient updating of large pretrained models across sites while preserving local governance, but ophthalmic evidence remains early (16, 29).

A recent example is SynthFed, a privacy-preserving federated framework for long-tail multi-hospital ophthalmic diagnosis that integrates VQ-VAE and GPT-based image synthesis to augment underrepresented classes in three-center fundus fluorescein angiography datasets (30). By generating minority-class synthetic images locally within a federated setting, the framework improved multi-disease classification performance, particularly for rare ophthalmic conditions, suggesting federated generative augmentation as a promising but early-stage strategy beyond conventional cross-silo classification.

**FIGURE 2:**
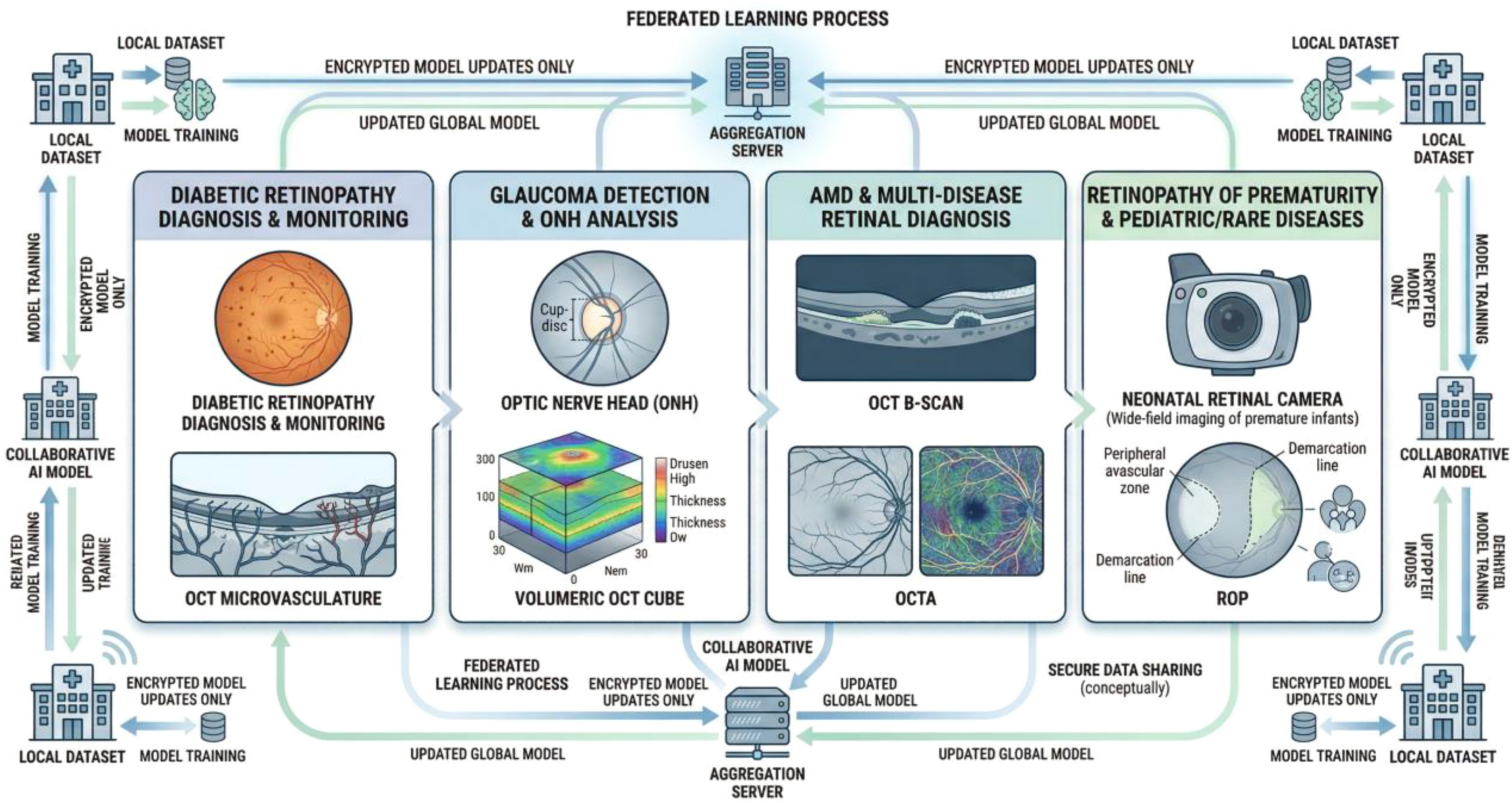
Disease-specific applications of federated learning across ophthalmology. Overview of major ophthalmic FL use cases, organized by disease and modality, including DR from fundus photography and OCT, glaucoma from volumetric OCT and multimodal screening data, AMD and multi-disease retinal classification from OCT/OCTA, and emerging use in pediatric ophthalmology and rare disease networks.

Across these disease areas, the evidence base is uneven. DR and glaucoma currently provide the strongest ophthalmology-specific examples of retrospective or multicenter FL evaluation, whereas AMD, OCTA-based analytics, pediatric retinal disease, foundation-model training, and federated generative augmentation are mostly supported by smaller, technical, or early translational studies. Readers should therefore distinguish benchmark performance from clinical utility and from readiness for real-world deployment. Representative ophthalmic FL applications are summarized in Table 1, and the disease-specific application landscape is shown in Figure 2.

**TABLE 1:** Representative applications of federated learning in ophthalmology.

Study/year	Ophthalmic task	Modality	Main FL approach	Privacy/security layer	Key translational message	Performance summary
Lo et al., (18)	DR classification and microvasculature segmentation	OCT/OCTA	Cross-silo FL	FL baseline	Demonstrated feasibility of collaborative OCT-based ophthalmic learning	Feasibility shown across OCT/OCTA tasks; no prospective clinical validation.
Ran et al., (23)	Glaucoma detection	Volumetric OCT	Multicenter FL	Privacy-preserving collaborative training	Landmark evidence that FL can approach centralized performance in glaucoma	Federated AUC 0.92 vs. centralized AUC 0.94.
Gholami et al., (25)	AMD diagnosis	OCT	FL + domain adaptation	Privacy-preserving distributed training	Highlighted importance of addressing domain shift	Diagnostic FL reported as feasible; domain shift remains a key determinant.
Nabil et al., (26)	Multi-disease retinal diagnosis	OCTA	Comparative FL strategies	FL baseline	Extended FL from single-disease tasks to broader ophthalmic classification	Performance varied by FL strategy and data distribution.
Yan et al., (27)	Ophthalmic FL platform	Mixed ophthalmic imaging	FedEYE platform	Workflow and orchestration support	Platform design is critical for translation	Platform-focused study; not primarily an accuracy benchmark.
Teo et al., (33)	Retinal imaging under adversarial risk	Retinal imaging	FL + blockchain	Blockchain-assisted security	Trust, tamper resistance, and adversarial resilience matter in real deployments	Security-focused study; evaluates adversarial resilience rather than standalone accuracy.
Nielsen et al., (11)	Privacy vulnerability analysis	Retinal imaging	Gradient-sharing FL	Attack analysis	FL updates can still leak image information, motivating stronger defenses	Attack study; demonstrates privacy leakage risk rather than clinical performance.
Gholami et al., (28)	Distributed foundation-model training	OCT	Self-supervised and domain-adaptive distributed learning	Distributed training paradigm	Ophthalmic FL is expanding toward foundation models	Distributed/foundation-model study; adjacent to FL but not a conventional cross-silo FL benchmark.

## Privacy, trust, and governance requirements for ophthalmic federated learning

### Federated learning is privacy-enhancing, not privacy-complete

A common misconception is that FL itself guarantees privacy because raw images remain local. In practice, gradients, weights, embeddings, or other shared updates may still reveal sensitive information. Leakage can involve not only direct patient identity, but also disease labels, demographic attributes, institutional membership, acquisition-device signatures, and latent biometric features embedded in retinal structure. Cross-domain evidence from handwriting, behavior, and visual-personality analysis similarly suggests that person-level attributes may remain recoverable from representations that appear task-specific; in ophthalmology, retinal images are especially sensitive because they may encode stable vascular and anatomic patterns. For clinically credible deployment, ophthalmic FL should therefore be understood as a layered trust model that combines collaborative training with explicit privacy protection, security monitoring, and governance safeguards (11).

### Differential privacy

Differential privacy (DP) adds calibrated noise to gradients, parameters, or outputs so that the contribution of any individual training example becomes statistically difficult to infer (8). In medical imaging, DP can reduce re-identification risk, although it usually comes with some loss of model utility. In ophthalmic FL, DP is especially relevant when explicit privacy accounting is required, such as in multicenter consortia or regulatory-sensitive collaborations, but authors should report the privacy budget and the associated performance trade-off rather than assuming that DP is clinically neutral.

### Secure aggregation and secure multi-party computation

Secure aggregation protocols are a practical baseline for production-oriented FL because they prevent the coordinating server from inspecting individual site updates in plaintext; only the combined update can be recovered (31). This is particularly relevant in ophthalmology, where many collaborations will rely on academic coordinating centers or cloud-supported consortia that are trusted operationally but still require formal privacy safeguards.

### Homomorphic encryption

Homomorphic encryption (HE) allows computation to be performed on encrypted values and is therefore attractive for FL aggregation (32). In ophthalmology, HE has already been explored in federated DR classification (22). Its main limitation remains computational overhead, particularly for OCT volumes, large models, foundation-model adaptation, or frequent training rounds.

### Blockchain, auditability, and trust infrastructure

Blockchain is not essential for every FL deployment, but it may add value when auditability, tamper resistance, and decentralized trust are priorities. In retinal imaging, blockchain-assisted FL has been proposed as a way to strengthen update traceability and improve resilience to adversarial attacks across institutions (33). The privacy and security techniques most relevant to ophthalmic FL are summarized in Table 2.

**TABLE 2:** Privacy and security techniques for ophthalmic federated learning.

Technique	What it protects	Strengths	Main limitations	Ophthalmic relevance
Differential privacy	Limits contribution of individual records	Formal privacy accounting	Accuracy trade-off; tuning complexity	Useful when privacy guarantees must be explicitly quantified
Secure aggregation	Hides individual client updates from server	Practical; scalable for cross-silo FL	Does not fully prevent all leakage or poisoning	Strong default layer for multicenter eye-hospital collaborations
Homomorphic encryption	Enables encrypted computation during aggregation	Strong confidentiality during aggregation	Computationally expensive; communication overhead	Attractive for high-governance DR and retinal collaborations
Secure multi-party computation	Prevents exposure of private inputs during joint computation	Strong cryptographic guarantees	Complex deployment; latency	Suitable for high-stakes multi-institution collaborations
Blockchain/audit ledger	Tamper resistance and update traceability	Transparency and accountability	Extra systems complexity; not always necessary	Useful for logging updates, governance, and adversarially sensitive consortia
Byzantine-robust aggregation	Defends against malicious or corrupted updates	Improves resilience to poisoning	May reduce efficiency or utility	Important where sites have uneven trust levels

### Threats: gradient inversion, membership inference, poisoning, and adversarial manipulation

**FIGURE 3:**
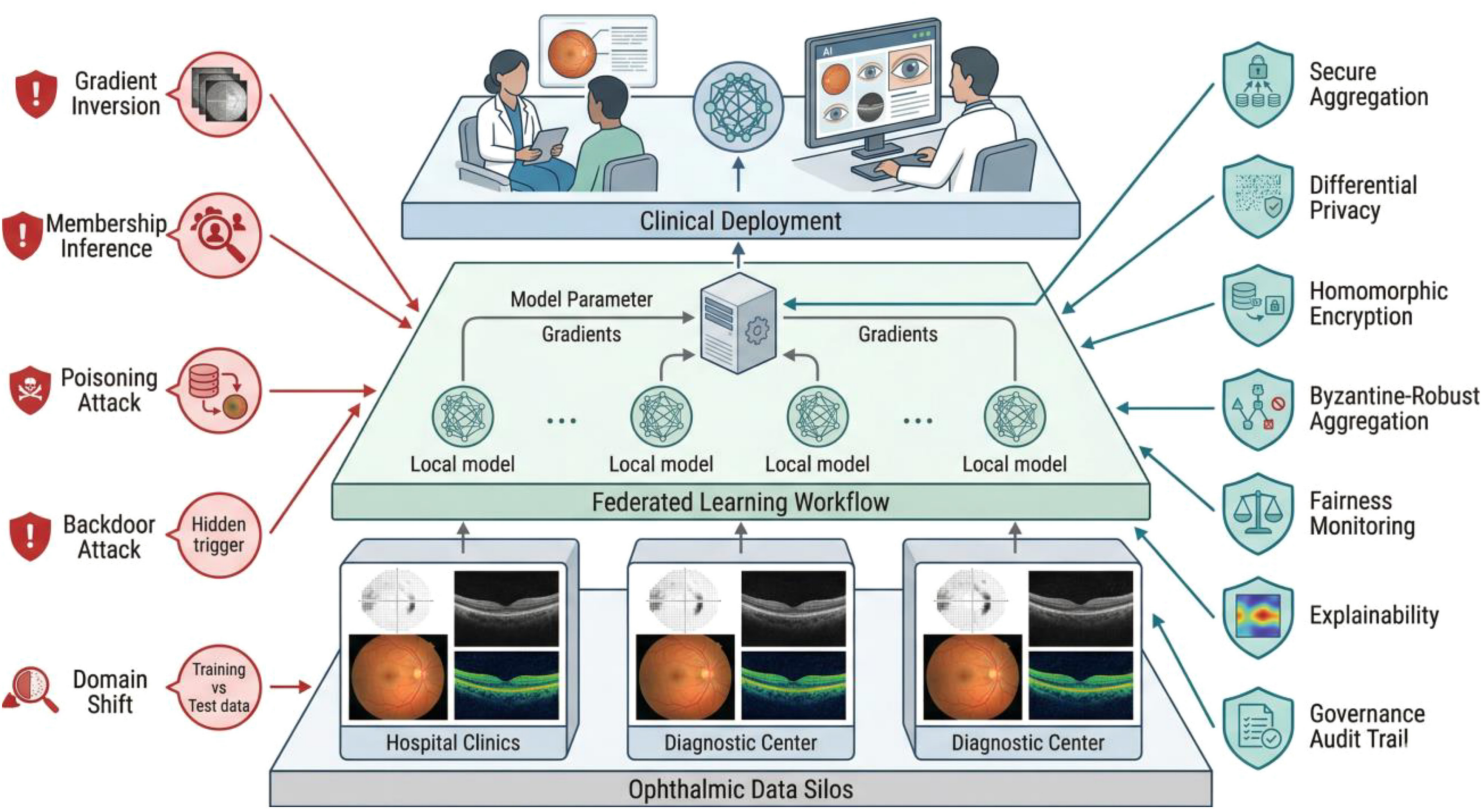
Threat model and defense stack for ophthalmic federated learning. Summary of the attack surface and layered defenses in ophthalmic FL. Threats include gradient inversion, membership inference, poisoning, backdoor updates, and site-level data heterogeneity. The corresponding defense stack includes secure aggregation, differential privacy, homomorphic encryption, robust aggregation, fairness monitoring, and audit/governance infrastructure.

Recent ophthalmic evidence shows that retinal image information may still be reconstructed from shared gradients under certain conditions (11). This challenges the assumption that FL alone is sufficient for patient protection. Threat modeling should distinguish reconstruction of image content, inference of disease labels, membership inference, institutional attribution, demographic inference, and recovery of latent biometric patterns. Other risks include backdoor attacks, label poisoning, and adversarially manipulated updates, all of which become more relevant as ophthalmic FL systems scale and become more connected to cloud-based infrastructure (33). The main attack surface and corresponding defense stack are illustrated in Figure 3.

## Barriers to real-world implementation in ophthalmic care

### Data heterogeneity and domain shift

Heterogeneity is arguably the defining challenge of ophthalmic FL. Imaging devices vary across vendors and generations, acquisition protocols differ between screening programs and tertiary centers, label distributions may be skewed, and referral thresholds reflect local practice patterns (14). Even within the same modality, OCT cube geometry, segmentation pipelines, and signal quality can differ markedly. For clinicians, this means that a collaborative model may appear robust overall while still underperforming in specific sites, devices, or patient groups.

Potential solutions include heterogeneity-aware objectives, domain adaptation, self-supervised pretraining, personalization layers, and generative replay (11, 30). Importantly, data harmonization should be viewed not as an alternative to FL, but as a prerequisite for clinically reliable collaboration and more interpretable cross-site validation (5).

### Communication efficiency and systems scalability

Communication is a practical bottleneck in FL, especially for large OCT volumes, transformer-based models, and foundation-model adaptation. Strategies such as model compression, quantization, sparse updates, asynchronous participation, and partial parameter sharing can mitigate communication cost. From an implementation perspective, however, communication efficiency is also a workflow issue: centers need systems that do not disrupt network operations, overburden local information technology teams, or delay model updates beyond clinically useful timelines (16).

### Fairness, equity, and under-represented groups

A privacy-preserving model is not necessarily an equitable model. Ophthalmic FL may inadvertently amplify bias if dominant sites contribute most of the training signal or if local disease distributions differ substantially from under-represented populations. Recent FL studies in medical imaging have begun to operationalize fairness metrics, but this agenda remains underdeveloped in ophthalmology (34). For clinical adoption, subgroup performance should be treated as a core safety issue rather than an optional secondary analysis.

### Explainability and clinical interpretability

For ophthalmic AI to influence real-world care, clinicians must trust not only aggregate performance but also case-level reasoning, failure modes, and the consistency of explanations across sites. Explainability in FL poses a special challenge because raw data cannot be centrally reviewed and site-specific saliency patterns may differ. Emerging work on fundus-based FL suggests that utility and explainability should be evaluated explicitly rather than inferred from centralized benchmarks alone (35).

### Interoperability, governance, and regulation

Translation of ophthalmic FL into clinical workflows requires more than technical feasibility. Institutions need shared expectations regarding data dictionaries, annotation standards, model cards, privacy accounting, audit trails, software validation, cybersecurity, and responsibility for post-deployment monitoring. Regulatory positioning will depend on jurisdiction and intended use: FL-trained ophthalmic AI may need to fit within FDA software-as-a-medical-device and predetermined-change-control expectations, EU Medical Device Regulation and AI Act requirements for high-risk AI systems, and China NMPA pathways for AI-enabled medical devices. These frameworks make version control, local validation, cybersecurity documentation, bias monitoring, and post-market surveillance central rather than optional. Ophthalmic FL will likely require alignment with broader medical data standards alongside ophthalmology-specific metadata conventions and harmonized labeling frameworks (4, 5). Key translational barriers and practical solutions are summarized in Table 3.

**TABLE 3:** Translational challenges and practical solutions.

Challenge	Why it matters in ophthalmology	Candidate solutions
Non-IID data	Device, population, and labeling differences are substantial	FedProx, personalization, domain adaptation, self-supervised pretraining
Communication burden	OCT volumes and large models increase bandwidth cost	Compression, quantization, sparse updates, asynchronous FL, adapter tuning
Privacy leakage	Gradients may reveal sensitive retinal information	Secure aggregation, differential privacy, homomorphic encryption, attack audits
Poisoning and adversarial attacks	Malicious updates can damage model reliability	Robust aggregation, validation rules, reputation systems, anomaly detection
Fairness	Dominant sites may bias global model behavior	Subgroup analysis, reweighting, fairness-aware FL objectives
Explainability	Clinicians need transparent decision support	Saliency auditing, site-level explanation reporting, prospective clinician validation
Governance and interoperability	Clinical deployment depends on shared standards	Metadata harmonization, protocol alignment, audit trails, data-governance templates

## Priorities for clinically deployable ophthalmic federated learning

### Multimodal federated ophthalmic AI

Future ophthalmic FL systems may combine fundus photographs, OCT, OCTA, visual fields, clinical text, systemic laboratory data, and longitudinal treatment histories. Multimodal FL may be especially valuable in glaucoma, diabetic eye disease, and inherited retinal disorders, where no single modality fully captures disease status. Although much of the broader multimodal federated AI literature remains outside ophthalmology, diabetes-care work illustrates how distributed multimodal data could support more equitable complication surveillance, including diabetic eye disease (29). Because ophthalmic vertical FL remains underdeveloped, future studies should report which modality or feature set is held by each site, how missingness is handled, and whether multimodal fusion improves external validation rather than only internal benchmark accuracy.

### Federated foundation models and parameter-efficient updating

The move toward ophthalmic foundation models creates both opportunity and complexity (36). Distributed training and federated adapter tuning may allow institutions to benefit from large-scale pretrained representations without full retraining or raw data transfer (28). However, distributed training should not be equated with clinically governed FL unless the study design includes client-level autonomy, raw-data locality, secure aggregation or equivalent safeguards, auditable update exchange, and cross-site validation under realistic governance constraints. Current evidence should therefore be read as promising but preliminary.

### Privacy-aware benchmarking in ophthalmology

The next generation of ophthalmic FL studies should benchmark not only discrimination metrics, but also privacy leakage, fairness, robustness, communication cost, interpretability, calibration, external validation, and deployment burden. Minimum reporting should include the number of institutions, sample size by site, imaging device distribution, data-partition strategy, model architecture, local baseline, centralized or pooled-data reference where ethically possible, confidence intervals, subgroup performance, external validation, and privacy-attack audit results. A model that performs well but remains vulnerable to gradient inversion, site-level dominance, or impractical maintenance requirements should not be considered clinically mature (34).

### Toward deployment-ready ophthalmic FL ecosystems

Ultimately, the field needs deployable ecosystems rather than isolated algorithms. This includes usable ophthalmic FL platforms, standardized study designs, external validation consortia, secure update auditing, governance templates that define data rights and responsibilities from the outset, and prospective evaluation in realistic care settings (27). No reviewed ophthalmic FL system can yet be considered broadly prospectively validated for routine clinical deployment. The long-term promise of FL in ophthalmology lies in enabling trustworthy collaboration at scale, not simply reproducing centralized performance under a new name (4).

## Limitations of this review

This article is a narrative review and has several limitations. The literature search was targeted rather than systematic, was limited to PubMed, Web of Science, Google Scholar, and reference-list screening, and did not use a registered protocol, formal duplicate-removal log, or PRISMA-style flow diagram. Inclusion was based on clinical and translational relevance, so selection bias is possible and some technical FL papers may not have been captured. Because study designs, disease tasks, imaging modalities, privacy layers, and evaluation metrics were heterogeneous, we did not calculate pooled estimates. The evidence base also remains dominated by retrospective, proof-of-concept, platform, or simulation studies, with limited prospective clinical validation. These limitations mean that the conclusions should be interpreted as a clinically oriented synthesis of the current landscape rather than as a quantitative estimate of FL effectiveness.

## Conclusion

Federated learning is a practical route to collaborative ophthalmic AI when raw data sharing is limited by privacy, governance, or operational constraints. Evidence across diabetic retinopathy, glaucoma, age-related macular degeneration, and OCT/OCTA-based applications suggests that FL can support multi-institutional model development, and selected studies show performance close to centralized training under controlled conditions. The field remains early: most systems are retrospective or proof-of-concept, prospective clinical validation is scarce, and FL does not by itself guarantee privacy, fairness, safety, or clinical readiness. Future work should prioritize transparent reporting, external and prospective validation, privacy-attack auditing, fairness analysis, regulatory planning, workflow integration, and post-deployment monitoring.
