# Supplementary material for: Federated learning for privacy-preserving ophthalmic artificial intelligence: clinical applications and translational challenges
Source: Front Med (Lausanne). 2026 Jul 29;13:1904004. doi: 10.3389/fmed.2026.1904004 (PMC13463149; doi:10.3389/fmed.2026.1904004)
Supplement: Supplementary file 1 [file Data_Sheet_1.pdf]

**Supplementary Table 1**. Positioning of the present narrative review relative to prior reviews

| Review / source | Publication scope | Primary focus | Gap addressed by the present review |
|---|---|---|---|
| Nguyen et al., 2022 | Ocular imaging FL literature to 2022 | Current progress and future directions for FL in ocular imaging | Updates the evidence base through March 2026 and adds explicit translational, privacy, and governance framing. |
| Lim et al., 2022 and Yang et al., 2024 | Privacy-preserving technologies in ophthalmology | Broad privacy-preserving AI and data-sharing issues | Focuses specifically on FL evidence, clinical disease areas, and deployment barriers. |
| Gulati et al., 2025 | Systematic review of federated deep learning for ocular disease imaging | Algorithmic and imaging-study synthesis | Adds clinical-readiness interpretation, regulatory context, and privacy-leakage distinctions. |

| | | | |
|---|---|---|---|
| Hallaj et al., 2025 | Glaucoma-focused FL review | Personalized learning, heterogeneity, and glaucoma collaboration | Places glaucoma evidence beside DR, AMD, OCTA, pediatric, platform, and foundation-model applications. |
| Present review | Ophthalmic FL literature and related privacy/governance work, January 2014 to March 2026 | Disease-specific clinical applications, privacy risk, and translation | Distinguishes benchmark performance from prospective clinical readiness and highlights field-level evidence gaps. |

**Supplementary Table 2**. Included representative ophthalmic federated learning studies and reason for inclusion

| Study / year | Study type or task | Reason for inclusion |
| --- | --- | --- |
| Lo et al., 2021 | OCT/OCTA microvasculature segmentation and DR classification | Early OCT-based ophthalmic FL application. |
| Karakaya et al., 2022 | Privacy-preserving DR detection | Fundus-based collaborative DR learning. |
| Matta et al., 2023 | Multicenter fundus DR screening network | Screening-network evidence for DR-focused FL. |
| Gholami et al., 2023 | OCT-based AMD diagnosis | Illustrates domain adaptation and site heterogeneity. |
| Tang et al., 2024 | Multi-disease ocular recognition | Ocular FL with domain adaptation across disease categories. |
| Ran et al., 2024 | Multicenter OCT glaucoma detection | Key quantitative example comparing FL and centralized training. |
| Yan et al., 2024 | FedEYE ophthalmic FL platform | Platform and workflow translation evidence. |

| | | |
|---|---|---|
| Teo et al., 2025 | Retinal imaging, blockchain, and adversarial risk | Security and auditability evidence. |
| Gholami et al., 2025 | Distributed ophthalmic foundation-model training | Adjacent evidence for decentralized large-scale representation learning. |
| Nielsen et al., 2025 | Homomorphic-encryption FL for DR | Cryptographic privacy layer in ophthalmic FL. |
| Chetoui and Akhloufi, 2023 | Vision Transformer-based FL for DR detection | Transformer-based DR FL example. |
| Nielsen et al., 2026 | Gradient-inversion attack analysis in retinal FL | Direct ophthalmic privacy-leakage evidence. |
| Nabil et al., 2026 | OCTA-based multi-disease ophthalmic diagnostics | Extends FL beyond single-disease fundus/OCT tasks. |
| Alam et al., 2026 | Asynchronous web-based OCT image analysis | Systems-oriented asynchronous FL example. |
| Penzel et al., 2026 | Fundus oculi datasets, utility, and explainability | Explainability and model-utility evaluation in FL. |
